# On the Diseases and Injuries of Seamen

**Published:** 1853-11

**Authors:** G. R. B. Horner

**Affiliations:** Surgeon U. S. Navy, Philadelphia


### *On the Diseases and Injuries of Seamen.* By G. R. B. Horner, M. D., Surgeon U. S. Navy, Philadelphia.

### *Rubeola, Parotitis, and Tonsillitis.*

During my whole term of active service, I have only known the crew of one vessel to be affected with measles, and that was the crew of the Delaware, who were infected at Norfolk just after she fitted out. The disease soon became epidemic, and was still lingering in her when I joined her three months after it first appeared. It principally attacked the boys, was accompanied with ordinary symptoms, fever, soreness of throat and eyes, but these were sometimes most affected, and remained inflamed for a long time. Indeed, so much ophthalmia preceded, accompanied and followed the measles, that they seemed to have tainted the eyes of many persons in a most extraordinary manner. For though the measles were without exception cured, yet the ophthalmia was the most obstinate ever met with by me. Some of the cases were perfectly incurable, the conjunctiva becoming granulated and chronically inflamed. Local and general depletion, astringent lotions of the greatest reputation, citrine ointment, the nitrate of silver, and all other remedies thought appropriate, were tried in vain. Consequently some of these cases had to be sent on shore before the ship sailed, and others to the United States after she arrived in Brazil. With the exception of these cases, I have very rarely failed in subduing ophthalmia by means of the medicines specified ; weak solutions of sup. acet. of lead, the nitrate of silver, and sulph. of zinc, warm bathing, cathartics, antimonials, blue mass in moderate quantities, and by occasionally using leeches and poultices to the eyes, and blisters on the nape of the neck. Setons also were employed in some cases of the worst kind with doubtful efficacy, and bathing with warm water in those of every class, as it was much more soothing than cold. The former lessens action, relaxes the inflamed parts, causes a free secretion from the meibomian glands and eyes, renders them more sensible to the action of cold, or astringent lotions, and thereby indirectly produces a tonic effect. When inflammation ran high in the above and like cases, scarification with a lancet on the inside of the lids was frequently performed. In some instances, however, it seemed to increase rather than diminish irritation, either from the great sensitiveness of the parts, or from an increase of tears having been caused to flow over the incisions.

For the cure of the measles, the remedies most prescribed were cooling drinks, seidlitz powders, and other gentle cathartics. Collyria, alum gargles, and brown mixture were given to the patients whose eyes, throats, or respiratory organs were much affected.

Into two of the ships mentioned another contagious disorder was introduced—the mumps, or technically speaking, cynanche parotidea. The first ship infected was the United States, and it was thought at New York, as she sailed from thence, though the disease appeared on the seventeenth day afterwards, and when she was two-thirds across the Atlantic. The period of incubation, therefore, was nearly three weeks, if not more, unless the disease originated on board, which is improbable. The first case was that of a man who had the usual symptoms and was cured of them, but seized with orchitis twelve days after he was taken under treatment. Another man had mumps in a reverse manner, the right testis having been tumefied and inflamed before he had the parotids sensibly affected, and the jaws made stiff. In a third case, the submaxillary glands were also enlarged, and as much, if not more so, than the parotids. Altogether, twenty cases happened, and the last one upon the 28th of August, that is sixty-three days after the appearance of the first case. Most of them were of a mild kind, but six were attended with orchitis of one or both testes, and the average cure was effected in seven and a half days. The youngest patient was seventeen years old, and the eldest thirty-eight years. Three more persons were above thirty, and ten were from seventeen to twenty-two years old. In the Savannah only ten persons had the disease; the youngest was a marine drummer of ten years, and the eldest a seaman of twenty-eight. Most of these cases were also of a mild character, and all were undoubtedly owing to contamination at New York, where the ship was when the first took place, though three did so at Boston, and four others after she sailed thence for the Pacific. In each vessel the disease seemed to continue until every person liable to it had been affected, for the last one in the United States was taken sick in the Archipelago, and the last patient in the Savannah when she had been at sea twenty-seven days, and was near Brazil. The average period of cure for the cases in the latter frigate was short of six days, although several of them, from the severity of the cold, were accompanied with tonsillitis.

The ordinary treatment was to give Epsom salts and tartar emetic in combination, to purge well. Sometimes the castor oil was given, or an emetic of ipecac. and tartarized antimony, when the testes were attacked. Afterwards the sick took the spiritus mindereri, or a solution of the last named medicine, rubbed the volatile liniment over the parotids, and wore hot linseed poultices over them. Their diet was low, their drinks cooling when fever ran high. In two cases this happened to such a degree as to demand free venesection, and in those where the parotids and testes were much inflamed leeches were applied. When the testes were attacked, blisters were occasionally substituted for volatile liniment to the parotids, and an evaporating lotion of water, alcohol and sup. acet. of lead were applied to the former instead of poultices. By the use of the above remedies all the cases were cured, but one of those in the United States was under treatment twenty-eight days, from being complicated with catarrh as well as orchitis. In the cases accompanied with tonsillitis, gargles had to be used, and I also, agreeably to my practice in that disease, lanced the tonsils freely. This very much shortened the treatment, and relieved the patient of pain and difficult deglutition, by disgorging the blood vessels and preventing swelling and suppuration. In fact, freely lancing the tonsils does in a few minutes what may require days or months to accomplish with medicines. It also prevents the part from remaining indurated and swelled after the inflammation has subsided, and saves thereby the necessity of excision. Were this practice generally adopted, in conjunction with appropriate medicines, the above operation would become almost obsolete. Certain it is, that I know of no instance of its having been required after I had treated tonsillitis—and many cases have been seen by me—of persons who had enlarged tonsils, ascribable to the neglect of incisions into them while inflamed. To make them, a lancet firmly fixed between the two sides of the handle was most used, but the blade is apt to turn to the right or left, and is too short to reach the glands unless the fingers holding the instruments are partly thrust with it into the mouth. For these reasons, a small straight scalpel is preferable, and the patient having had the lower jaw and tongue firmly depressed by means of a spoon handle or spatula, without being thrust so far back as to cause gagging, the point of the scalpel should be passed directly through the projecting portions of the glands. By doing this, a copious flow of blood will be obtained without danger of puncturing the external walls of the fauces and cutting the internal carotid arteries in their passage to the base of the cranium. Such an accident could only happen from great carelessness,, awkwardness, or ignorance of the anatomy of the parts, and is not more liable to occur than that of leeches—recommended by some—getting into the stomach or larynx, either before or after application. These reptiles likewise are very disgusting and horrifying to many patients, even when they are externally applied, and few would submit to their being introduced into their throats. This difficulty might be obviated by their being used externally, but they would be less efficient and would have to be applied in larger numbers, and this would be very objectionable where they are scarce and the patients poor, or the leeches very dear, as at Lima, at which city they cost a dollar a piece by the hundred. At sea, too, it is difficult to keep a supply of them; often they cannot be had for any price, and for these, if for no other reasons, it is preferable to employ incisions. But though these possess such efficacy, they were not relied on alone, were frequently not required, and always assisted by medicines. The gargles mentioned were used, likewise some made of the nitrate of potash dissolved in sweetened water, in the proportion of ʒi. or ʒij. to ℥vij., and others of Cayenne pepper, molasses, and vinegar, and of like proportion. In the majority of cases, however, a gargle of ʒij. of powdered borax, ℥i. of honey and ℥viij. of strong sage tea was found the most pleasant and useful, and most prescribed. In severe cases antimonials, and other diaphoretics, cathartics, frictions over the throat, and hot pediluvia were used. Hot poultices applied externally to the affected part after friction with volatile liniment and turpentine or some other stimulating one, afforded much relief, and the same benefit may be derived from filling a small bag with wood ashes, wetting them with warm ley, and fastening the bag about the patient's throat while he is reposing. This is a domestic remedy which I have seen used in country practice, but a very good one, from its combined fomenting and stimulating properties, which promote perspiration, and divert irritation from the fauces to the integuments.

